# Enhancing Multisensory Experience in CAVE Virtual Reality Through Olfactory Sensing

**DOI:** 10.3390/s26123910

**Published:** 2026-06-19

**Authors:** Vasilis Vasileiadis, Anastasios Theodoropoulos, George Lepouras

**Affiliations:** 1 HCI-VR Lab, University of the Peloponnese, 22131 Tripoli, Greece; dit2401cst@go.uop.gr (V.V.); ttheodor@uop.gr (A.T.); 2 Department of Performing and Digital Arts, University of the Peloponnese, 21100 Nafplio, Greece

**Keywords:** olfactory virtual reality, multisensory experiences, olfactory displays, virtual reality, CAVE, human–computer interaction

## Abstract

The integration of olfactory feedback into Virtual Reality (VR) applications remains significantly underexplored compared with other sensory modalities, particularly within room-scale Cave Automatic Virtual Environments (CAVEs), where related research is even more limited. To address this gap, this paper presents *Scentree*, a custom olfactory system capable of delivering scents in real time based on user interactions, along with *Smelling Ancient Greece*, an olfactory-enhanced VR experience developed for integration within our CAVE system. Central to the proposed approach is the concept of the *Diegetic Olfactory Feedback Loop*, which reframes olfaction from a passive ambient effect into an active, interaction-driven feedback mechanism embedded within the narrative context of the virtual environment. To evaluate the system, we conducted a technical performance assessment and an exploratory user study (N=51) examining participant perceptions of immersion, presence, perceived realism, usability, and overall user experience. The findings support the feasibility of interaction-driven olfactory feedback as a multisensory design approach for CAVE environments and provide a foundation for future controlled investigations of olfactory feedback in immersive VR.

## 1. Introduction

Virtual reality (VR) systems are still seeking to improve the levels of multisensory accuracy, aiming to increase the sense of presence, immersion, realism, and engagement. Immersive experience design has overwhelmingly been dominated by visual, auditory, and haptic modalities because of their efficiency [1,2]. Olfaction, despite its promising results [3,4,5,6,7], remains underexplored.

Among these sensory modalities, olfaction occupies a particularly promising position. The olfactory system is uniquely connected to the limbic system, enabling scent to evoke strong emotional responses and autobiographical memories with a directness that other sensory modalities cannot replicate [4,7]. Research has demonstrated that olfactory cues can improve context-dependent memory recall [3], enhance the sense of presence and immersion in virtual environments [5,6], and contribute to the perceived realism and emotional authenticity of immersive experiences. Furthermore, multisensory integration research indicates that combining congruent stimuli across modalities produces perceptual benefits that exceed those achievable through any single modality alone [8], suggesting that the thoughtful integration of olfactory cues within VR could meaningfully strengthen the overall multisensory experience.

Recent comprehensive reviews of OVR display technology [9] reveal a growing body of work spanning diverse delivery mechanisms and a range of application domains. However, these reviews also highlight persistent technical challenges that continue to constrain the practical deployment of olfactory feedback in interactive systems: activation latency, scent cross-contamination, limited intensity control, and the difficulty of spatially targeting aromas within larger physical spaces. These challenges become considerably more acute when olfactory systems are considered for deployment within room-scaled immersive environments, where the physical scale, ventilation conditions, and multi-user nature of the space introduce design requirements that existing OVR systems are not equipped to meet.

The majority of OVR systems described in the literature have been designed for either head-mounted display (HMD) configurations or single-user desktop setups [9]. Cave Automatic Virtual Environments (CAVEs) [10] represent a fundamentally different class of immersive system, in which visual content is projected onto the surrounding walls and floor of a room-scale enclosure, enabling multiple users to share the virtual space simultaneously without individual head-mounted displays. This architecture offers several distinctive advantages: it supports co-located multi-user participation, eliminates HMD-related ergonomic concerns such as motion sickness and discomfort during extended sessions, and allows surrounding observers to engage with the experience in real time. These properties make CAVEs a compelling platform for public-facing installations, shared educational experiences, and collaborative VR applications. Yet despite these advantages, the integration of olfactory feedback within CAVE environments has received very limited attention in the research literature, and the existing body of work provides little guidance on how olfactory systems should be architecturally designed, technically evaluated, or experientially integrated within game-engine-driven CAVE applications [9].

This paper addresses this gap through three primary contributions. First, we present *Scentree*, a portable, modular, and fully reproducible olfactory feedback system, designed for real-time, interaction-triggered scent delivery within a CAVE environment via seamless integration with the Unity game engine. The system supports four independent olfactory channels, each housed in a detachable scent container, enabling scalable and cross-contamination-aware scent delivery. Second, we introduce *Smelling Ancient Greece*, a proof-of-concept olfactory-enhanced VR experience deployed within our CAVE (*MobiCAVE*) system [11], in which participants explore a virtual museum exhibition spanning four thematically distinct areas, each mapped to a contextually appropriate scent. Third, conceptually, we propose the *Diegetic Olfactory Feedback Loop* as a principled design framework that repositions olfaction from a passive ambient effect into an active, interaction-driven feedback modality—one in which scent becomes a direct and meaningful consequence of user agency within the virtual environment, grounded in enactive theories of perception [12] and embodied interaction [13].

To evaluate the proposed system and experience, we conducted both a technical performance assessment and an exploratory user study (N=51), structured around three research questions: (RQ1) Does the olfactory system operate reliably, responsively, and as intended within the virtual environment? (RQ2) Is interaction-driven olfactory feedback associated with positive perceptions of immersion, presence, and realism? (RQ3) Is the interaction model intuitive, understandable, and easy to use within a shared CAVE environment? In addition to descriptive statistics, we report internal consistency estimates, inter-item correlation analyses, and inferential analyses examining the relationship between prior VR experience and user outcomes—providing a more comprehensive psychometric characterization of the evaluation instrument than is typical in exploratory OVR studies.

The remainder of this paper is organized as follows. Section 2 reviews related work on olfactory VR displays, covering desktop, HMD-based, and CAVE-specific approaches. Section 3 describes the system architecture, hardware design, firmware, virtual experience design, and user study methodology. Section 4 reports the technical evaluation results, descriptive statistics, reliability analyses, and inferential findings. Section 5 discusses the implications of these results in relation to multisensory integration theory and interaction design. Section 6 outlines the limitations of the present work and directions for future research. Section 7 concludes the paper.

## 2. Related Work

### 2.1. Early Innovations

The introduction of digital scent technologies (DST’s) began with a company called Digiscents, which created a gadget called Ismell. The apparatus was made up of pots filled with scented oil [14]. These oils were evaporated by heating a mixture of pots in accordance with the code sent from the computer. The heated vapors were then expelled by a fan.

Another company, Aromajet, created a prototype aroma dispenser device known as Pinoke. This tiny gadget could be worn or placed in front of the desk and deliver scents to the user [14]. The same company later developed a device called the Scentkiosk scent dispenser. This device aimed for a more personalized approach, allowing users to create their own fragrances from a combination of other fragrances the cartridges held.

Trisenx, another company, introduced a beta version of their scent dome technology [14]. Scent dome’s cartridge system contained twenty scent cartridges, making it capable of storing and releasing up to twenty different odors. These early innovations introduced technologies that enabled computer-controlled scent delivery. Even though they were limited to desktop applications, had slow response times, and required exclusive hardware, they established the basis for later developments for OVR (Olfactory Virtual Reality) displays.

### 2.2. OVR Displays for Virtual & Desktop Applications

Olfactory Virtual Reality (OVR) displays can be categorized based on three parameters [9]. These parameters include ( 1) delivery methods, (2) presentation approaches and (3) application areas.

Delivery methods refer to the procedure where the odor vaporizes and is transferred to the user. Delivery methods include three techniques, which are: (1) delivery via heat, (2) delivery via airflow and (3) delivery via atomization.

The technique of heat-based delivery simulates the natural distribution of scents by vaporizing scent molecules with heat and releasing them as a soft plume. Airflow delivery is achieved by directing air currents to transport smell molecules towards the user. Lastly, the atomization technique is the process of utilizing ultrasonic vibrations to break liquid components into droplets of mist.

Based on their presentation approach OVR displays are categorized as ubiquitous, handheld and wearable.

Ubiquitous devices frequently depend upon fixed installations, like diffusers in a room that enable the production of ambient smells. Handheld devices let people engage directly with fragrances. Those devices are usually attached to a VR controller so that the user can redirect the controller to their nose to smell. Lastly, wearable OVR devices incorporate scent emitters into individual gadgets such as necklaces.

**Figure 1:**
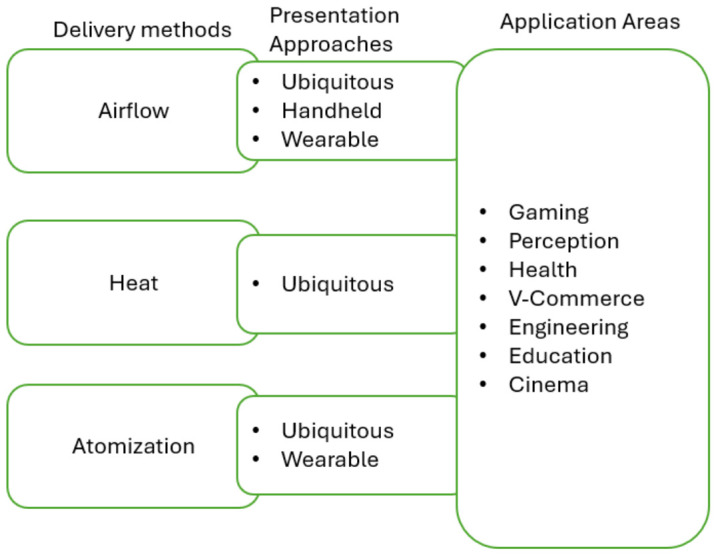
Classification of Olfactory Virtual Reality Displays.

Finally, olfactory virtual reality displays are categorized based on their application area, reflecting the context in which these displays are deployed. Previous research has identified seven key areas, including (1) Gaming, (2) VR cinema, (3) Health, (4) Education & Training, (5) V-Commerce, (6) Perception studies and (7) Engineering. This classification is shown in Figure 1 below:

A plethora of olfactory displays have been developed to enable olfaction in virtual immersive environments. Ubiquitous devices have concentrated on delivering multiple scents in the environment. Ranasinghe et al. [15] explored the integration of olfactory feedback in their horror game Tainted, where scents were used to communicate narrative information, signal in-game events, and evoke emotional responses. Their system consisted of an Arduino Mini Pro connected to four ultrasonic mist generators, each containing a vial of fragrance solution. A fan mechanism dispersed the emitted scents into the environment. The game was developed in the Unity engine and communicated with the Arduino through digital output signals, enabling real-time activation of the mist generators during gameplay. The system successfully delivered context-specific odors in synchronization with gameplay events, demonstrating the feasibility of real-time olfactory feedback in interactive environments. However, several limitations emerged. To maintain consistency with the game’s narrative, the researchers selected only floral and fruity fragrances, which reduced participants’ ability to distinguish between scents during prolonged exposure. Despite this limitation, the synchronized use of multiple scents allowed olfactory stimuli to function as an additional feedback channel alongside visual and auditory cues. Their findings indicated that olfactory feedback enhanced emotional engagement, increased immersion, and was perceived as more informative than alternative sensory cues, thereby contributing positively to the overall user experience. At the same time, participants reported difficulties in differentiating between similar aromas over extended periods, highlighting challenges related to scent clarity and perceptual discrimination. Overall, the study demonstrates both the potential of olfactory feedback to enrich multisensory VR experiences and the practical limitations associated with scent distinguishability.

Another study by [16] developed an olfactory display that can deliver up to twenty-four distinct fragrances. Their system uses an Arduino mega that controls four servo MG996R motors to press sprays from modular fragrance spray bottles, enabling simultaneous odor release from four channels. The Arduino is also connected with a L298N motor driver which controls two 12 V fans. Those fans are used to direct the scent to the user or rotate backward to clean the air and avoid cross-contamination. Their system is scalable and can be expanded by adding more servo motors and scents, while also preventing the need for reprogramming. They also created a C# application for Unity integration, enabling scent distribution via different events. Their limitations include cross-contamination with scents that were released within a small time gap, lack of files for automated scent triggering and insufficient control over the persistence times and conflicts between various scents.

Researchers [17] have recently explored olfactory interaction in virtual reality through the development of handheld olfactory displays integrated with virtual reality systems such as the HTC Vive. In their study, a graspable olfactory display was evaluated through an immersive virtual reality wine-testing simulation, where users identified individual and blended scents across multiple trials. Their results show that participants quickly adapted to the olfactory system, demonstrated consistent performance between sessions, and experienced increased difficulty as scent mixtures became more complex. Furthermore, users demonstrated exploratory and realistic smelling behaviors, such as adjusting their sniffing distance and mimicking real wine-tasting gestures, suggesting a high level of immersion and intuitive interaction. However, the study also highlights limitations, including a small sample size, short-term evaluation, and perceptual imbalances in scent mixtures, as well as usability issues related to hardware constraints.

A contrasting approach to handheld olfactory displays is presented by wearable systems integrated into VR headsets. Wearable olfactory displays are devices designed to be worn by the user or integrated directly into head-mounted displays (HMDs) aiming to deliver scents close to the user’s nose. These systems are typically lightweight, compact, and portable.

A study by Zhang et al. [18] proposed a configurable framework for real-time scent prediction and delivery in video games. Their approach utilized computer vision and cross-modal analysis to trigger olfactory stimuli without requiring prior integration into the game’s source code. The system adopted a pipeline-based architecture that combined screen capture, player input, and natural language prompts (via CLIP) to detect in-game events and release corresponding scents through a wearable device. The framework was evaluated within a Minecraft scenario through a user study involving 30 participants. The findings revealed that contextually congruent scents were significantly preferred over random scent delivery or conditions without olfactory feedback, suggesting that semantic consistency between the virtual environment and the emitted odors plays an important role in enhancing user experience. However, the observed improvements in immersion were limited, with statistically significant effects appearing primarily in measures of attention and focus. This indicates that automated scent triggering alone may not be sufficient to support deeper forms of multisensory engagement. Overall, the study highlights both the potential of scalable and domain-independent olfactory integration systems and the importance of meaningful interaction design in achieving stronger perceptual and experiential impact within immersive environments.

Similarly, ref. [19] developed a wearable olfactory device in the form of a necklace called “Essence”. The device uses a piezoelectric transducer to vaporize oils that are stored in a small container that is connected to a cotton filter. To keep it compact, most of the electronic components were placed in the back of the necklace. The front part consists of the piezoelectric transducer, the 7 mL container and the cotton wick. The back part holds the microcontroller (an AT-mega32u4), a 3.7 V Lithium battery that powers the device for 27–28 h by defaulting the bursts at a rate of 1 s of scent every 20 s to ensure the previous odors have faded and a Bluetooth Low Energy board (BLTE) for wireless communication. The device can also emit scents based on context data from the smartphone, such as GPS and date and time. They also experimented with the release of odors based on biometric data such as Heart rate, electrodermal activity, and brain activity. They used the E4 wristband to measure the heart rate and electrodermal activity and the EEG MUSE Headband for brain activity. “Essence’s” limitations include the limitation of a single scent at a time, odor refills, habituation and desensitization and the limits of frequency and intensity of scent.

In the same context a study by de Paiva Guimarães et al. [20] developed an olfactory display attached to an HMD, using a Raspberry Pi–controlled ultrasonic atomizer to deliver scents, synchronized with virtual events. In their user study, participants exposed to a floral aroma during a VR experience reported significantly higher levels of perceived immersion, pleasantness, and satisfaction compared with a control group where no scent was received. Their results demonstrate that even the addition of simple olfactory stimuli can positively influence user experience and presence in virtual environments. However, the study also presents several limitations: the interaction was passive and event-triggered rather than exploratory, only a single scent was used, and a notable proportion of participants in the control group reported perceiving odors despite none being delivered. Furthermore, the system lacked detailed control over intensity and personalization, thus limiting its use in more complex or adaptive olfactory interactions.

### 2.3. Olfactory Displays for CAVE Environments

Recent research [21,22,23] has tried to integrate olfactory stimuli in CAVE environments. Duong et al. [21] conducted a large-scale user experiment (N = 551 N=51) in an omnidirectional dome-based virtual environment, where participants experienced a simulated chocolate store under different sensory conditions. Using both self-reported measures and psychophysiological data, their study revealed that the increase in the number of sensory modalities had led to significantly higher enjoyment, arousal, perceived sensory intensity, and more positive store and brand evaluations. These findings suggest that olfactory cues, particularly when combined with other modalities, enhance the overall experiential richness and emotional engagement of virtual environments. However, the study presents notable limitations: immersion and presence were not directly measured, the olfactory stimulation was limited to a single scent delivered via a diffuser, and the experimental design did not explore factors such as sensory congruency or intensity variation. As such, while the results provide indirect support for the role of olfaction in enhancing user experience in CAVE systems, they do not offer conclusive evidence regarding its effect on immersion.

In addition, Shin et al. [22] have investigated the addition of olfactory stimuli in CAVE-like environments in the context of environmental restoration rather than immersion directly. In their study, they examined whether virtual windows with views of nature could enhance psychological recovery in a bustling virtual cafe setting using a CAVE-type projection system. Participants were exposed to windowless, closed-window, or open-window conditions while simultaneously experiencing consistent auditory stimuli, visual projections, and olfactory stimuli such as the aroma of coffee and additional scents associated with nature. Their results, based on the Perceived Recovery Scale (PRS) and the Rehabilitation Outcome Scale (ROS), show that both open-window and closed-window conditions significantly improved perceived restoration compared with the initial windowless condition, particularly in dimensions such as allure and the feeling of being “away” from daily demands. However, the addition of olfactory and auditory cues of nature did not yield significant improvements beyond visual manipulation alone. While this study demonstrates that multisensory enhancement can enhance perceived immersion in CAVE-type environments, it also highlights that olfactory stimuli may not necessarily add any measurable benefits relative to visual information in terms of rehabilitation outcomes and that immersion itself was not directly assessed using presence-based measures.

Another study by Quintana et al. [23] investigated whether exposure to fear-related body odors could influence the formation of interpersonal impressions through emotional transfer. Participants were immersed in a virtual bar environment, where they were exposed to fear, joy, or neutral odors derived from sweat and delivered via a respirator mask. Their results show that exposure to fear-related odors significantly increased self-reported anxiety compared with neutral and joyful conditions. Furthermore, the mediation analysis revealed that increased anxiety reduced trust in a virtual character, showing that olfactory stimuli can significantly alter social evaluation processes in immersive environments. Nevertheless, the study did not explicitly measure immersion or presence. On the contrary, it focused on emotional and interpersonal outcomes, and the absence of physiological data due to technical issues limits the reliability of its multimodal validation. Overall, the findings suggest that olfactory stimuli in CAVE-type virtual reality systems can significantly alter emotional state and social cognition, even without explicit measures focused on immersion.

A vast number of olfactory virtual reality (OVR) displays have been developed for virtual reality and desktop applications, each presenting noticeable technical approaches for scent generation and control. The plethora of those devices has been designed either for HMD or desktop environments, where scents are limited to the emission of only a single scent at a time and are restricted to only a single user. In addition, the number of studies that tried to integrate olfactory displays into CAVE systems are very few and the existing literature does not present us with the effects of olfactory stimuli on user experience in CAVE scenarios. Thus, the aim of this study is to examine the impact of olfactory cues when we transition olfaction from an ambient enhancer into an active diegetic feedback loop.

## 3. Materials and Methods

### 3.1. System Architecture

The proposed system provides a portable and fully reproducible solution for integrating scent diffusion into the *MobiCAVE* environment. Its primary purpose is to deliver olfactory feedback based on user interactions within the virtual environment. Since CAVE environments differ from traditional HMD and desktop-based VR systems due to their larger spatial scale and support for multi-user interaction, a different design approach is required. The system can be positioned anywhere within the *MobiCAVE* environment without requiring structural modifications. Although it can diffuse up to four scents simultaneously, the use of detachable scent containers enables support for an unlimited number of scents. In addition, the system can be easily integrated into projects developed using the Unity 3D (version 2022.3.60f1 LTS, Unity Technologies, San Fransisco, CA, USA) game engine.

At the functional level, the system establishes a connection between digital interactions and scent emission in the physical environment. When users interact with a virtual object, Unity transmits a character command to the Arduino via serial communication. The Arduino receives the signal and determines which piezoelectric transducer to activate, subsequently enabling the corresponding relay. Since each piezoelectric transducer driver board includes a built-in button for controlling scent emission, the relay module emulates button presses rather than directly switching the power supply. More specifically, the relay performs a sequence of three simulated button presses to alternate between emission and idle states. This approach ensures reliable state transitions between the piezoelectric transducers and enables immediate scent emission following user interaction with a virtual object.

The system architecture is divided into two coordinated subsystems: the hardware subsystem and the software subsystem. The hardware subsystem consists of an Arduino Uno R3, responsible for communication with the Unity 3D environment and relay activation, a relay module that emulates button presses to activate the corresponding piezoelectric transducers, and four piezoelectric transducers housed within detachable scent containers for scent diffusion. Each piezoelectric transducer is connected to an individual driver board powered via USB through a USB hub. The USB hub can either be powered directly through the PC’s USB port or, as in our implementation, through an external power supply. The software subsystem includes Unity components that:

1. Ensure communication with Arduino.
2. Read Interactions and send signals to the Arduino for interpretation.
3. Arduino’s firmware (Activation Protocol).

**Figure 2:**
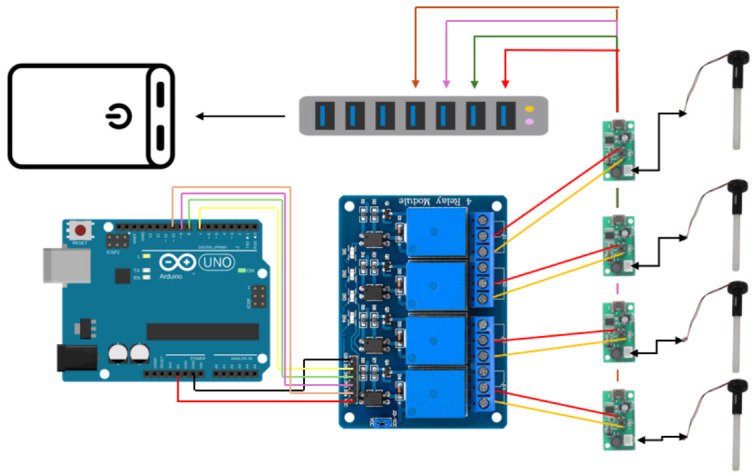
*Scentree’s* Architecture.

A high-level diagram summarizing the architecture is shown in Figure 2 below:

### 3.2. Hardware

*Scentree* is constructed using a set of low-cost components that together form a fully modular and portable scent delivery system. The core control component is an Arduino Uno R3, which is responsible for receiving serial commands from Unity and interpreting these signals to activate the corresponding relay channels. The Arduino is connected to a four-channel 5 V relay module, where each relay channel emulates a physical button press used to control an ultrasonic piezoelectric transducer.

Four ultrasonic piezoelectric transducers operate as the system’s scent emission units. Each transducer consists of a driver PCB and an ultrasonic atomization module, which are responsible for converting scented liquid into mist. To ensure independent operation and minimize cross-contamination between scents, each piezoelectric transducer is housed within a separate detachable container containing its own scented solution. In order to enable external control without interfering with the driver board’s power regulation circuitry, each driver board was modified by soldering two jumper wires to the button contact pads. These wires were then connected through the relay module’s COM/NO ports, allowing the relay to emulate physical button presses automatically. This modular architecture provides scalability, as supporting additional scents only requires the addition of more piezoelectric transducer modules, relay channels, and detachable scent containers.

**Figure 3:**
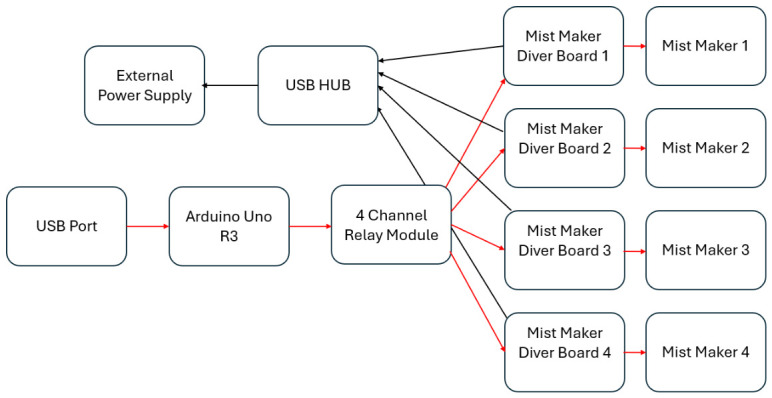
Wiring Diagram.

The piezoelectric transducer driver boards are powered through a multi-port USB hub connected to a 10,000 mAh external power supply. The inclusion of an external power source increases the portability of the system while also creating electrical isolation between the piezoelectric transducers and the Arduino-relay circuitry. This separation helps prevent higher current loads from affecting the microcontroller’s operation. The Arduino is powered independently through a USB connection to the computer running the Unity application. Collectively, these components form a compact, flexible, and reproducible system capable of delivering controlled real-time scent diffusion based on user interactions with virtual objects. A summary of all components, plus their specifications, is shown in Table 1, as well as a wiring diagram in Figure 3.

### 3.3. Mechanical Design

**Figure 4:**
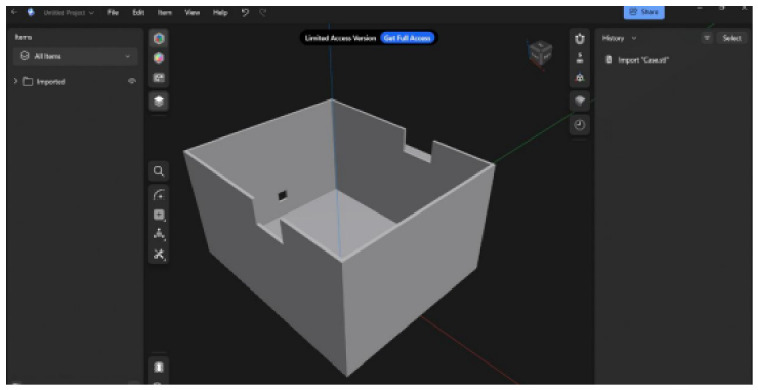
*Scentree’s* Case model created in the Shapr3D software.

**Figure 5:**
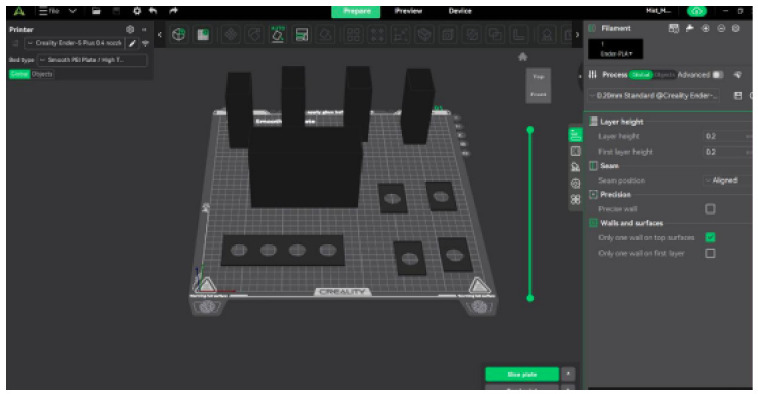
*Scentree’s* parts edited in Creality Slicer software for 3D Print.

To enhance the portability, safety, and reproducibility of the olfactory system, a dedicated enclosure was designed and fabricated using additive manufacturing techniques. The enclosure was modeled in Shapr3D, (version 26.100) exported as STL files, and prepared for fabrication using Creality Slicer, (version V7.0.0.4212) before being manufactured on a Creality Ender 5 Plus (Shenzhen Creality 3D Technology Co., Ltd., Shenzhen, China) 3D printer. Figure 4 shows *Scentree’s* case model in the Shapr3D software, while Figure 5 demonstrates all the parts inside the Creality Slicer software.  

The enclosure was designed to accommodate all hardware components while preserving the modular nature of the system. In addition to providing structural support, it secures the electronic components, wiring, and scent diffusion modules within a unified housing. This protective design reduces the risk of accidental contact with exposed circuitry and cables during installation, demonstrations, and experimental sessions, making it particularly suitable for deployment in public-facing CAVE environments.

A key design objective was to simplify assembly and maintenance procedures. To achieve this, the enclosure utilizes a snap-fit construction, eliminating the need for additional fasteners during assembly. This approach enables rapid installation and disassembly while facilitating routine tasks such as refilling scent containers, cleaning components, or replacing hardware modules. Furthermore, the snap-fit mechanism ensures consistent positioning of internal components, contributing to more reliable and repeatable hardware configurations across experimental sessions.

By combining modularity, protection, and ease of maintenance, the enclosure improves the practical deployment of the system and supports the reproducibility of future implementations. The digital design files can be readily reproduced, modified, and adapted for alternative hardware configurations or deployment scenarios.

### 3.4. Firmware

The firmware developed for the Arduino implements a byte-oriented serial interface operating at 9600 baud over USB, enabling real-time communication with the Unity engine. The program maps incoming ASCII characters in the range “1”–“4” to the relay module’s four channels through the Arduino’s digital pins “D7”–“D10”, which correspond to the relay module’s input pins “IN1”–“IN4”. This mapping allows each signal transmitted from Unity to immediately trigger the corresponding relay channel, thereby emulating a button press on the associated piezoelectric transducer driver board.

During initialization, all relay pins are set to a HIGH state to maintain the system in an idle configuration. The serial interface is then initialized, followed by a short two-second delay to allow the USB host connection to complete enumeration. Finally, a diagnostic message is printed to confirm that the system has initialized successfully.

Within the main execution loop, the firmware continuously monitors the serial receive buffer. When a valid character between “1” and “4” is detected, the firmware converts the received value into a zero-based index and invokes the corresponding channel-specific activation protocol. This activation protocol performs a sequence of three simulated button presses that emulate the physical button interaction required by the piezoelectric transducer driver board. This approach ensures reliable state transitions regardless of the device’s previous operational state. The sequence proceeds as follows:

1. The first press activates the piezoelectric transducer and initializes odor emission.
2. The second press stops emission after a short period.
3. The third press returns the piezoelectric transducer into idle mode.

Constants are set at the beginning of the sketch, setting our time parameters:

- PRESS_DURATION = 500 ms (time pressed).
- ON_TIME = 5000 ms (emission duration after the first press).
- INTER_PRESS_DELAY = 300 ms (time gap between the second and third press).

This firmware design provides deterministic control over each piezoelectric transducer through a simple serial communication protocol, ensuring repeatable operation and reliable timing behavior. By relying on fixed timing parameters, the system can be easily reconfigured to accommodate different experimental conditions and interaction scenarios. The firmware was developed using the Arduino IDE (version 1.8.19) and is fully compatible with any ATmega328P-based microcontroller.

To perform the automated system evaluation described in Section 3.6, the same activation protocol was extended with an additional logging mechanism. Before executing the three-press activation sequence, the firmware initializes and increments an internal test counter while recording the test start time using the millis() function. After the activation sequence is completed, the firmware calculates the total execution duration and transmits both a start and end message through the serial connection. These messages include information such as the test identifier, channel identifier, and measured execution duration. This logging extension does not modify the timing parameters or alter the behavior of the activation protocol; instead, it provides additional diagnostic data to support further system evaluation and performance analysis.

### 3.5. Arduino-Unity Communication

As mentioned earlier, communication between Unity and the Arduino is established through a serial USB connection operating at 9600 baud using Unity’s built-in .NET package, System.IO.Ports. The project was configured to use the .NET API compatibility level to ensure that the SerialPort class is available during both compilation and runtime. This serial interface enables the Unity 3D game engine to transmit single-character ASCII commands that directly control *Scentree* in real time. Algorithm 1 below shows Arduino’s main logic.
**Algorithm 1** Pseudocode of Arduino’s main logic**Require:** Relay Module Pins = Arduino Digital Pins[7, 8, 9, 10] →[IN1, IN2, IN3, IN4]**Require:** PRESS_DURATION = 500 ms, ON_TIME = 5000 ms, INTER_PRESS_DELAY = 300 ms  1:**procedure** Setup  2:    **for** *i* ← 0 to 3 **do**  3:        Configure relayPins[*i*] as OUTPUT  4:        Write HIGH to relayPins[*i*]  5:    **end for**  6:    Initialize serial at 9600 baud  7:    Wait 2000 ms  8:    Log “Arduino ready”  9:**end procedure**10:**function** Loop11:    **while** true **do**12:        **if** serial has data **then**13:           command ← read one character14:           **if** command ∈ {’1’,’2’,’3’,’4’} **then**15:               index ← command − ’1’16:               Log “Activating piezoelectric transducer” + (index + 1)17:               ActivateThreePress(index)18:           **end if**19:        **end if**20:    **end while**21:**end function**22:**procedure** Button Emulation(index)23:    pin ← relayPins[index]24:    PressButton(pin, PRESS_DURATION)25:    Wait ON_TIME26:    PressButton(pin, PRESS_DURATION)27:    Wait INTER_PRESS_DELAY28:    PressButton(pin, PRESS_DURATION)29:**end procedure**30:**procedure** PressButton(pin, duration)31:    Write LOW to pin32:    Wait duration33:    Write HIGH to pin34:**end procedure**

Within the Unity environment, a dedicated script named Arduino_Listener, attached to an empty GameObject, is responsible for initializing and maintaining the serial connection. During startup, the script opens a predefined serial port configured at 9600 baud, logs the connection status, provides a method for transmitting character commands to the serial interface, and properly closes the connection when the application terminates.

The same serial communication interface is also utilized by the AutoTester script within the automated evaluation environment described in Section 3.6. The AutoTester periodically sends trigger commands through the Arduino_Listener while simultaneously listening for timing messages transmitted by the Arduino firmware. These messages are subsequently recorded into a locally stored CSV file containing the performance data used for the quantitative evaluation of the system. The results of this evaluation are presented in Section 4.1.

At the same time, interactive objects serve as an intermediary between user actions and hardware activation through the Arduino_Listener script that is assigned to them. Furthermore, another dedicated script named Interactive_Object is assigned to objects that are meant for scent emission. This script exposes a public int set as mistMakerId (1, 2, 3, 4) and listens for key presses so that when the correct key is pressed, it executes a raycast forward of the camera up to an adjustable distance. If the ray hits the object, the script sends the ID as a single-character ASCII command to the Arduino via the Arduino_Listener. This request then results in the relay, so it activates the corresponding piezoelectric transducer, initializing the activation protocol, which executes the button presses, thus starting scent emission. Algorithm 2 depicts the Arduino-Unity communication logic.
**Algorithm 2** Unity Arduino Listener Logic**Require:** portName = “COM5” (Hardcoded), baudRate = 9600  1:serialPort ← null  2:**procedure** Start  3:    ConnectToArduino  4:**end procedure**  5:**procedure** ConnectToArduino  6:    **try**  7:    serialPort ← NewSerialPort(portName, baudRate)  8:    SetReadTimeout(serialPort, 50)  9:    SetWriteTimeout(serialPort, 50)10:    Open(serialPort)11:    **if** IsOpen(serialPort) **then**12:        Log(“Connected to Arduino on” + portName “COM5”)13:    **end if**14:    **catch** e15:    LogError(“Failed to connect:” + e.Message)16:**end procedure**17:**procedure** SendCommand(command)18:    **if** serialPort ≠ null **and** IsOpen(serialPort) **then**19:        **try**20:        Write(serialPort, command)21:        Log(“Sent command:” + command)22:        **catch** e23:        LogError(“Serial write error:” + e.Message)24:    **else**25:        LogWarning(“Serial port not open!”)26:    **end if**27:**end procedure**28:**procedure** OnApplicationQuit29:    **if** serialPort ≠ null **and** IsOpen(serialPort) **then**30:        Close(serialPort)31:    **end if**32:**end procedure**

**Figure 6:**
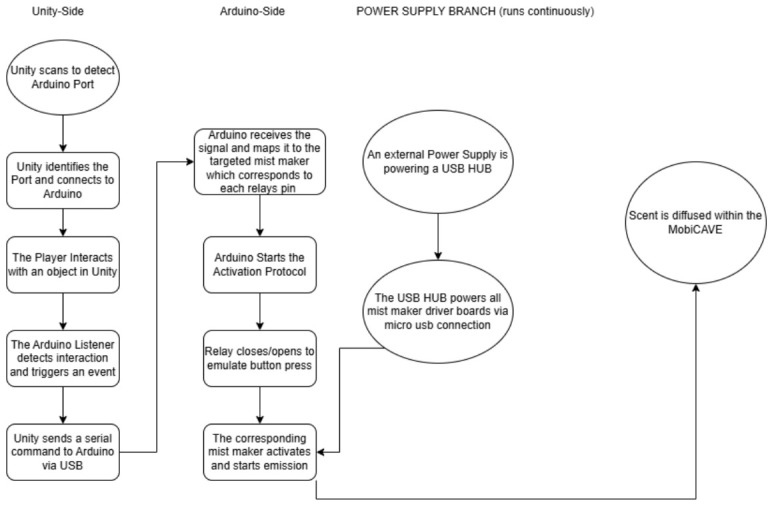
Process Flowchart.

This implementation follows a simplified asynchronous request model: Unity transmits a single-byte command for each interaction, and Arduino automatically runs the full activation protocol before going back to the idle state. The serial port opens once, only during scene initialization, and closes when the application is terminated. Furthermore, the port Arduino is using is hardcoded, meaning that no scanning occurs during execution, which results in the connection with the microcontroller being established. Figure 6 below demonstrates a flowchart of the whole process until emission.

### 3.6. Performance Evaluation

To evaluate the system’s quantitative indicators of reliability and time behavior, we created a dedicated test environment in Unity. This environment consists of a simplified scene and four interactive objects that are mapped to each of the four physical scent channels that are controlled by the Arduino-relay subsystem. To create automatic multiple iterations, we created a dedicated AutoTester script that triggers presses without user intervention. Algorithm 3 demonstrates how interactive objects work, while Figure 6 demonstrates a flowchart of the whole process.

During each trial, the AutoTester script periodically transmitted a single-character ASCII command requesting the activation of a specific channel. Upon receiving the command, the Arduino executed the activation protocol and measured the total duration of the activation sequence using the millis() function. For each trial, the firmware transmitted both a start marker and an end marker containing the trial identifier, channel identifier, and measured activation duration. Unity, through the Arduino_Listener component, continuously monitored the serial port, parsed the incoming messages, and recorded them into a locally stored CSV file. This file contained the Unity timestamp, trial ID, channel ID, event type, and total activation duration in milliseconds.

The Unity test environment was configured to perform a total of 200 activations, corresponding to 50 trials per channel, with a one-second pause between consecutive trials to simulate a realistic experimental pace. The resulting dataset was subsequently processed in Excel to compute the trial success rate, as well as the mean, standard deviation, and overall range of activation durations for each channel. In addition, a one-way ANOVA was conducted to determine whether statistically significant differences existed in the mean activation times across the four channels. These quantitative results are presented in Section 4.1.
**Algorithm 3** Interactive _Object Logic**Require:** arduinoListener (2), mistMakerId (1–4)**Require:** interactDistance = 3.0, interactKey = Key.E  1:playerCamera ← null  2:**procedure** Start  3:    playerCamera ← GetMainCamera()  4:    **if** playerCamera is null **then**  5:        LogError(“No MainCamera found!”)  6:    **end if**  7:    **if** mistMakerId < 1 **then**  8:        mistMakerId ← 1  9:    **end if**10:    **if** mistMakerId > 4 **then**11:        mistMakerId ← 412:    **end if**13:**end procedure**14:**procedure** Update15:    **if** GetKeyDown(interactKey) is true **then**16:        ray ← NewRay(playerCamera.position, playerCamera.forward)17:        **if** Raycast(ray, out hit, interactDistance) is true **then**18:           **if** hit.collider.gameObject is this.gameObject **then**19:               **if** arduinoListener is not null **then**20:                   command ← ConvertToString(mistMakerId)21:                   arduinoListener.SendCommand(command)22:                   Log(“Interacted with cube (ID “+ mistMakerId +”)”)23:               **else**24:                   LogWarning(“ArduinoListener not assigned on” + this.name)25:               **end if**26:           **end if**27:        **end if**28:    **end if**29:**end procedure**

### 3.7. Virtual Experience Design

#### 3.7.1. Virtual Experience Overview

To further investigate the role of olfactory feedback within a multisensory virtual environment, we developed a proof-of-concept application titled *Smelling Ancient Greece*, implemented in the Unity game engine and deployed within our CAVE system, *MobiCAVE*. The experience takes the form of a virtual museum exhibition designed to combine visual, spatial, and olfactory stimuli within a coherent narrative framework.

The virtual environment consists of four thematically distinct areas inspired by different aspects of ancient Greek culture:

- The Baths.
- The Gymnasium.
- The Symposium.
- The Altar.

Users can freely navigate the environment, explore exhibits, and interact with selected virtual objects following an exploratory interaction model. The experience was designed to support co-located multi-user participation, allowing multiple users to share the environment simultaneously without requiring individual head-mounted displays.

To support multisensory coherence, each thematic area combines visual context, spatial layout, contextual information, and olfactory cues. As users approach selected exhibits, on-screen textual descriptions are displayed, providing contextual information related to the object or environment being observed. Technically, these descriptions are triggered through collider-based proximity checks attached to predefined exhibit objects. This mechanism reinforces the narrative framing of each space while contextualizing the corresponding olfactory stimuli.

Each thematic area is mapped to one of the four physical scent channels of the *Scentree* system, establishing a direct relationship between the virtual environment and the associated olfactory output. This mapping enables users to associate specific locations with corresponding scents, strengthening perceptual consistency across sensory modalities.

**Figure 7:**
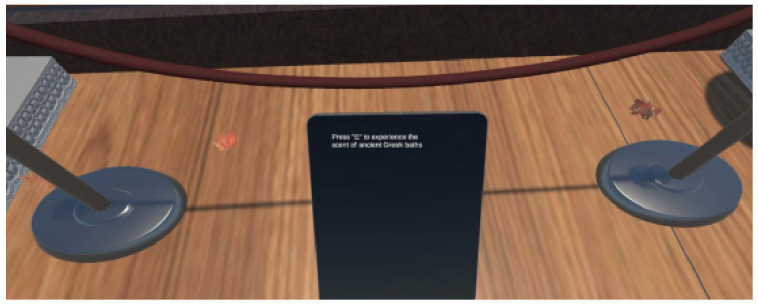
The Interactive kiosk that serves as the interactive object responsible for scent activation.

In contrast to passive scent diffusion approaches, olfactory activation within the experience is designed as an explicit user-driven interaction. To ensure reliable operation in a shared environment and prevent unintended activations, scent emission is triggered through interaction with designated virtual kiosks, implemented as interactive virtual objects placed within each thematic area. Each kiosk corresponds to a specific olfactory channel and functions as the sole activation point for that zone. Figure 7 presents the interactive kiosk.

On the implementation level, kiosk interaction is handled through the Unity scripts Arduino_Listener and Interactive_Object. When a user activates a kiosk, the corresponding channel identifier is transmitted as a single-character ASCII command through the Unity-Arduino serial interface described in Section 3.5. The Arduino then interprets the received command and executes the predefined activation protocol, which emulates button presses on the corresponding piezoelectric transducer driver board and initiates scent emission.

**Figure 8:**
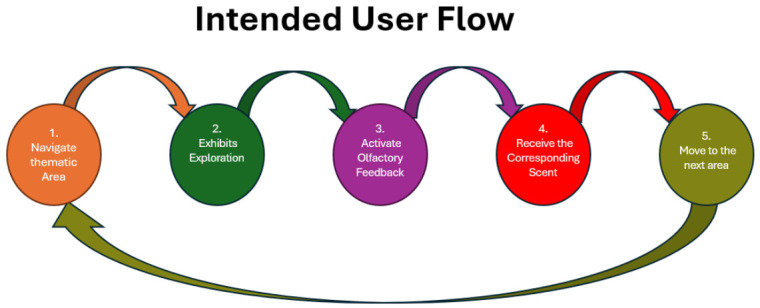
The Intended User Flow.

The interaction process was designed as a cyclical workflow that encourages users to explore multiple thematic areas while engaging with the olfactory system. As shown in Figure 8, users (1) navigate a thematic area, (2) explore exhibits to review contextual information, (3) activate the corresponding kiosk and (4) receive scent feedback, before (5) moving to the next area to start over.

Beyond the interaction logic, the museum layout was also designed around the physical constraints of scent diffusion within a shared CAVE environment. In particular, the spatial arrangement aimed to minimize cross-contamination between scents while supporting a predictable reset period during which residual odors could dissipate before users entered another thematic area.

To achieve this, the virtual museum was organized into four spatially distinct scent zones, each corresponding to a single olfactory channel and containing its own dedicated interactive kiosk. The kiosks were intentionally positioned within their respective thematic areas so that scent activation occurred only after users had already entered the intended narrative context.

**Figure 9:**
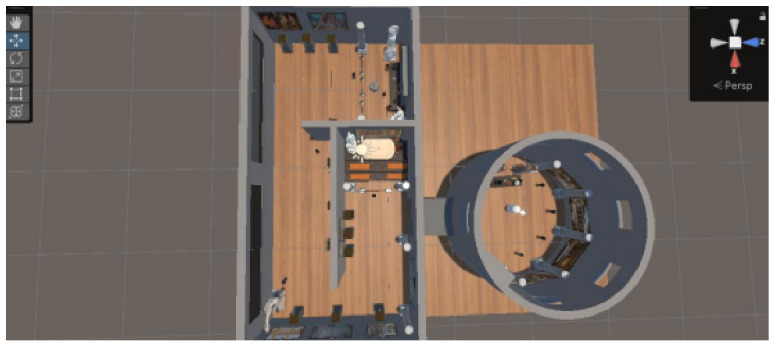
Top-down view of the “Smelling Ancient Greece” Unity Museum layout, showing the spatial arrangement of themed areas and the separation between zones to support controlled scent triggering and reduce cross-contamination.

Transition spaces between thematic areas functioned as neutral zones without olfactory interactions or scent-triggering events. These areas were designed (as shown in Figure 9) to reduce accidental activations during movement and to provide spatial separation that allowed residual odors to dissipate before users encountered a different scent stimulus. Since calculating precise fluid dynamics and spatial diffusion in a room-scale CAVE is highly complex, our approach to managing scent persistence and cross-contamination relied on architectural spatial design. By utilizing neutral transition spaces, we allowed natural air dissipation to act as a buffer, mitigating the blending of aromas between different interactive zones. As a result, the placement of exhibits, entrances, walls, and interaction points was treated as part of the multisensory design strategy rather than solely as an aesthetic or navigational consideration.

The selection of scents was guided by their semantic association with each thematic context. Scents related to relaxation, physical activity, social interaction, and ritual practices were selected to reflect the intended atmosphere of each area. By aligning olfactory cues with the environmental and narrative context, the experience aims to strengthen perceived realism, immersion, and multisensory coherence within the virtual environment.

#### 3.7.2. Diegetic Olfactory Feedback Loop

The experience is structured around a *diegetic olfactory feedback loop*, in which scent functions as an integrated component of the virtual environment rather than as a passive ambient effect. In this framework, olfactory stimuli are directly tied to narrative context and user interaction, forming a closed interaction cycle in which user actions dynamically trigger context-dependent olfactory responses.

The loop is theoretically grounded in the enactive approach to perception [12], which states that perception is not a passive reception of sensory input but is enacted through the organism’s active engagement with the environment. In the context of VR, presence is sustained when user actions produce appropriate, apparent, and expected consequences in the virtual world [24,25]. By shifting from passive ambient scenting to interaction-driven “enactive smelling”, olfactory feedback becomes a direct and meaningful consequence of user agency.

To analytically describe this closed interaction cycle, we formalize the diegetic olfactory feedback loop as a discrete-time state transition model. Let *t* denote the discrete interaction step. We define:

- $U_{t}$: The user’s perceptual and cognitive state at time *t* (e.g., sense of presence, expectations, spatial understanding),
- $I_{t}$: the user’s deliberate interaction at time *t* (e.g., activating a virtual kiosk or object),
- $O_{t}$: the olfactory stimulus emitted at time *t*,
- $V_{t}$: the synchronized visual/audiovisual stimulus at time *t*.

When the user performs an interaction $I_{t}$ within a given environment state $E_{t}$, the system computes and emits the corresponding olfactory response through a mapping function *f*:

$$O_{t}=f\left(E_{t},I_{t}\right).$$

In practice, *f* is implemented by the Unity-Arduino control logic, which maps specific interaction events and contextual states to the activation of particular scent channels in *Scentree*.

The user’s perceptual state is then updated through multisensory integration, modeled by a function *g* that combines prior user state, olfactory feedback, and audiovisual information:

$$U_{t+1}=g\left(U_{t},O_{t},V_{t}\right).$$

Here, *g* represents the cognitive process by which the user integrates olfactory, visual, and auditory cues into an updated spatial functional mental model of the environment [24,25]. The closed-loop interaction can thus be summarized as:

$$E_{t}\to I_{t}\to O_{t}\to U_{t+1}\to E_{t+1},\;$$

where $E_{t+1}$ reflects the updated virtual context as experienced and interpreted by the user after olfactory feedback and multisensory integration have occurred.

**Figure 10:**
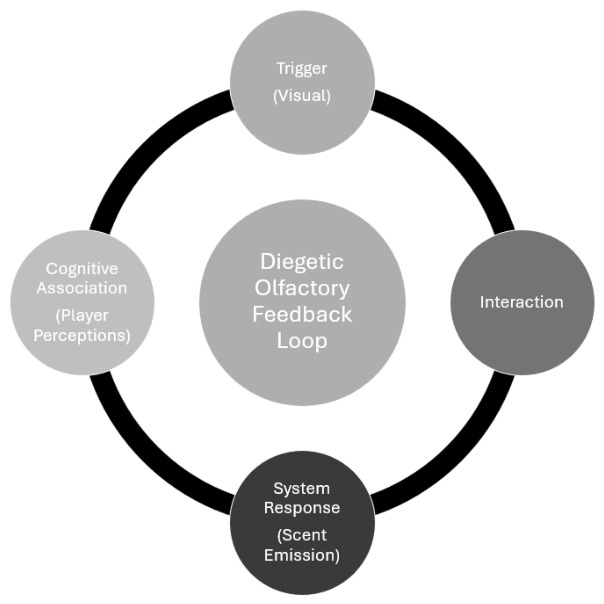
The Diegetic Olfactory Feedback Loop.

This formalization distinguishes the proposed approach from traditional ambient scenting techniques, where odors are passively diffused without direct user control or explicit coupling to interaction events. In our model, olfaction is treated as an active feedback modality that responds deterministically to user actions and contextual state, supporting stronger perceptual integration and enhancing the coherence of the multisensory experience. Through this closed-loop structure, olfactory feedback contributes not only to immersion and presence but also to spatial understanding and contextual awareness, positioning scent as a principled component of interaction design in CAVE environments. Figure 10 demonstrates the diegetic olfactory feedback loop.

### 3.8. Integration Within the MobiCAVE Environment

#### The *MobiCAVE* System

*MobiCAVE* [11] is an interactive Virtual Reality CAVE (Cave Automatic Virtual Environment) system designed to support immersive shared experiences. The system presents a simplified yet effective implementation of CAVE technology that is both portable and suitable for mobile and public exhibition scenarios. The *MobiCAVE* structure consists of a surround-screen projection environment with a diameter of 3.5 m and a height of 3 m. The environment incorporates 15 ultra-slim monitors arranged in a borderless configuration with 0.9 mm bezels, mounted on a custom-built aluminum truss structure. The side monitors can be configured in either a 120-degree or 90-degree orientation, allowing adaptation to different spatial requirements.

The system is powered by a high-performance workstation equipped with multiple synchronized graphics processing units, enabling all 15 displays to operate simultaneously from a single computer. A high-resolution projector mounted at the top of the truss structure projects visuals onto the floor using a specialized reflective surface. At the same time, a depth-sensing camera supports motion capture for position tracking, spatial mapping, object detection, and full-body tracking. Audio immersion is achieved through a surround sound system integrated into the environment.

The design of *MobiCAVE* emphasizes scalability for room-scale environments, usability under varying lighting conditions, and support for multi-user collaboration and shared participation, allowing attendees to both observe and actively engage with the experience. In addition, the system prioritizes portability through simplified installation and uninstallation procedures suitable for field deployments and traveling exhibitions. The platform is also designed for accessible maintenance, reduced operational cost, energy efficiency, and low-noise operation to facilitate communication between participants, while maintaining safety in both its structural and electrical systems.

**Figure 11:**
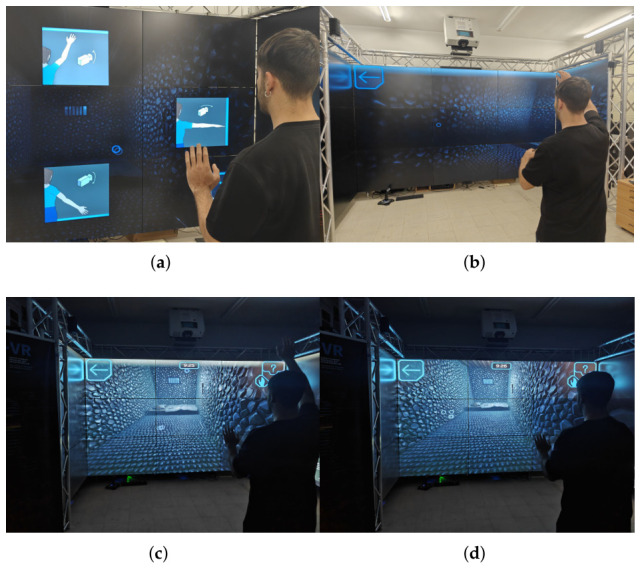
User interacting with the *MobiCAVE* system across different lighting conditions: (**a**,**b**) demonstrate interaction with bright lightning; (**c**,**d**) demonstrate interaction in the dark.

In contrast to traditional VR setups that rely exclusively on head-mounted displays, *MobiCAVE* supports a range of interaction modalities, including full-body interaction through motion capture systems. Depending on the application scenario, users may interact either through body movements, gesture-based navigation, or external input devices. This flexibility enables rapid user transitions during public exhibitions while also allowing surrounding attendees to observe and participate in the experience in real time, fostering a shared and socially engaging immersive environment. The *MobiCAVE* environment is shown in Figure 11 below:

### 3.9. User Study Methodology

To further evaluate the impact of olfactory feedback on multisensory virtual reality experiences, a user study was conducted with 51 voluntary participants. The participant sample consisted of 42 male and 7 female participants, while one participant identified as non-binary/other and one preferred not to disclose their gender. The study was conducted within the *MobiCAVE* environment and aimed to investigate the role of interaction-driven olfactory cues on user experience in terms of immersion, presence, and perceived realism. In addition, the study evaluated the operational reliability and perceived responsiveness of *Scentree*, as well as usability and interaction challenges associated with the proposed diegetic olfactory feedback loop within a shared CAVE environment.

The evaluation was structured around the following research questions:

1. **RQ1: System Validation & Technical Efficacy** Does the olfactory system operate reliably, responsively, and as intended within the virtual environment?
2. **RQ2: Immersion, Presence & Realism** Is interaction-driven olfactory feedback associated with positive perceptions of immersion, presence, and realism?
3. **RQ3: Usability & Interaction Design** Is the interaction model intuitive, understandable, and easy to use within a shared CAVE environment?

Before initiating the experience, participants completed a pre-experiment questionnaire collecting demographic information, prior familiarity with VR systems, previous exposure to olfactory-enhanced VR experiences, self-reported sensitivity to scents, and potential factors affecting olfactory perception. Anonymous participant identifiers were used to associate pre- and post-experiment responses while preserving participant anonymity.

Participants then explored the *Smelling Ancient Greece* virtual museum exhibition within the *MobiCAVE* environment. During the experience, users navigated through the four thematic areas, interacted with exhibits, and triggered olfactory feedback using a shared controller. This interaction design ensured that scent delivery remained directly associated with deliberate user actions rather than passive environmental diffusion, while it also allowed for rapid user changes while at the same time avoiding the additional calibration procedures typically required for motion-capture-based interaction.

Following the experience, participants completed a post-experiment questionnaire consisting of Likert-scale items (1–5) and open-ended responses. The questionnaire was designed to evaluate four primary dimensions:

1. **System Performance and Technical Reliability** Questions examined perceived synchronization, responsiveness, reliability, scent intensity, and timing consistency of the olfactory system.
2. **Immersion, Presence, and Realism** Questions assessed perceived immersion, engagement, sense of presence, and the extent to which the integration of visual and olfactory stimuli contributed to the realism and coherence of the experience.
3. **Usability and Interaction Design** Questions evaluated the clarity, intuitiveness, and ease of interaction with the kiosk-based scent activation mechanism.
4. **User Experience & Acceptance** Questions examined effects on the overall user experience and the acceptance of the integration of olfactory feedback in CAVE environments.

Several questionnaire items were adapted from established and validated VR evaluation instruments. Measures of presence, immersion, and involvement were derived from the Presence Questionnaire (PQ) [24], the Igroup Presence Questionnaire (IPQ) [25], and the presence constructs described by Usoh et al. [26] and Slater [27]. Usability-related items were adapted from the System Usability Scale (SUS) [28] and the Technology Acceptance Model (TAM) [29]. Items related to multisensory integration, memory, and perceptual coherence were informed by validated multisensory research, including Dinh et al. [30] and Shams and Seitz [8].

Because no standardized psychometric instrument exists for olfactory VR, all scent-specific items (e.g., scent intensity, latency, comfort, and contextual appropriateness) were adapted from the validated olfactory evaluation framework presented by Ranasinghe et al. [31], which provides established criteria for assessing olfactory delivery performance in interactive systems. These items therefore follow validated engineering-based evaluation dimensions rather than psychometric constructs.

Together, these sources ensure that each construct in Table 2 is grounded either in a validated VR presence/usability instrument or in a validated olfactory VR evaluation framework. Although the study relies on subjective participant reports, this approach aligns with standard practice in VR research, where presence, immersion, realism, and multisensory coherence are inherently experiential phenomena. As Schubert et al. [25] note, “although immersion is objectively quantifiable, presence—or, more precisely, the sense of presence—is a subjective experience and only quantifiable by the user experiencing it.” Consequently, subjective measures are not only appropriate but necessary for assessing experiential constructs in immersive environments.

To facilitate the analysis and interpretation of the questionnaire data, individual survey items were grouped according to the validated constructs presented in Table 2. Specifically, items IPR1–IPR4 were used to assess immersion and presence, while IPR5–IPR7 evaluated perceived realism. System Validation and Technical Efficacy was assessed through items SYS1–SYS7, Usability and Interaction Design through items USA1–USA4, and User Experience and Acceptance through items UX1–UX5. For each construct, descriptive statistics were calculated by averaging the corresponding item responses across participants. The results are presented according to the three research questions defined in the methodology, with questionnaire constructs mapped to the relevant research objectives.

**Figure 12:**
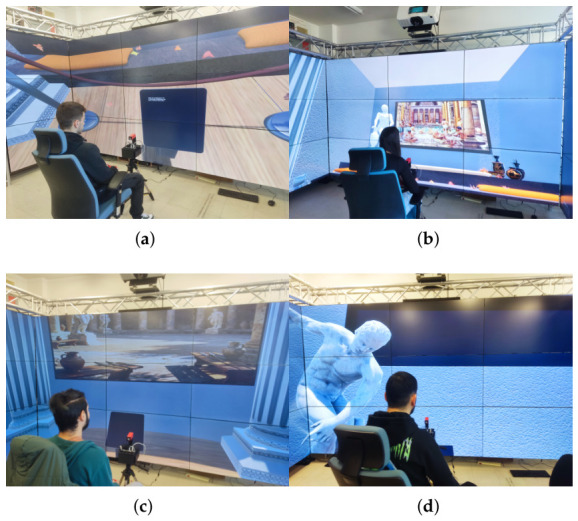
Users interacting with the *Scentree* system within the *MobiCAVE*: (**a**) User is interacting with the kiosk interface to activate *Scentree*; (**b**) User is navigating through the virtual exhibition; (**c**,**d**) Demonstrate *Scentree* delivering the corresponding aroma to the users.

In addition to subjective questionnaire responses, objective system-level timing measurements collected from the automated testing environment and Arduino logs were used to support the technical evaluation of synchronization accuracy and operational reliability. Each experimental session lasted approximately 10–15 min, including briefing, interaction with the virtual environment, and questionnaire completion. Figure 12 demonstrates users interacting with the *Scentree* olfactory system and the *Smelling Ancient Greece* virtual museum exhibition within the *MobiCAVE*.

More specifically, the evaluation examined whether explicitly triggered olfactory feedback, integrated within the narrative and spatial context of the virtual environment, contributed to a coherent and intuitive multisensory interaction experience. The study was therefore designed to investigate olfactory feedback not as a passive atmospheric effect but as an active interaction modality embedded within a diegetic interaction loop.

## 4. Results

### 4.1. System Evaluation Results

In Unity’s automated test environment, the system performed a total of 201 tests on the four aroma channels. Approximately 50 trials were performed on each channel except channel 3, in which 51 trials were performed after the Arduino reset. Nevertheless, we decided to include this trial since its timing coincides with those of the others on the same channel. Across all channels, the full activation sequence—including the three simulated button presses, odor emission period, and inter-press delays—had an average duration of 6800.692 ms (SD = 0.724 ms), demonstrating highly consistent timing behavior. Given the nominal protocol duration of 6800 ms, these results demonstrate highly accurate and repeatable timing performance. The averages and standard deviations per channel are presented in Table 3.

To determine whether there were any consistent timing variations between channels, a one-way analysis of variance (ANOVA, single factor) was conducted. The ANOVA resulted in $SS_{\mathrm{between}}=0.8023$ and $SS_{\mathrm{within}}=104.0733$, with $df_{\mathrm{between}}=3$ and $df_{\mathrm{within}}=197$, resulting in F(3,197)=0.50622 and p=0.678432. Since p>0.05, there are no statistically significant differences in the mean activation duration between the four channels, validating that all outputs have statistically equal time performance.

### 4.2. User Study Results

The results are presented according to the three research questions defined in the methodology.

For RQ1 (System Validation and Technical Efficacy), participants perceived the olfactory system as reliable and well synchronized with the virtual experience. Odor diffusion was rated as highly synchronized with virtual events (M=4.47, SD=0.70), while reports of noticeable delays or mismatches were low (M=1.90, SD=1.20). Participants also agreed that scents dissipated adequately before subsequent activations (M=3.67, SD=1.29), suggesting that the timing before moving to the next interactive kiosk was generally effective in limiting residual odors. Furthermore, scent intensity was considered appropriate (M=4.35, SD=0.69), and participants largely disagreed that the aromas were unpleasant or excessively strong (M=1.98, SD=1.10). These subjective assessments are consistent with the objective timing measurements reported in Section 4.1, indicating that *Scentree* operated with a high degree of accuracy and reliability.

Regarding RQ2 (Effects on User experience), participants reported a strong overall multisensory experience. Immersion was rated highly (M=4.14, SD=0.78), while presence scores (M=4.02, SD=0.84) indicated a substantial sense of “being there” within the virtual museum. Perceived realism received the highest ratings (M=4.49, SD=0.61), reflecting that the integration of visual and olfactory stimuli was considered coherent and believable. Participants also reported a strong correspondence between scents and their associated locations (M=4.25, SD=0.84), suggesting that the selected aromas were contextually appropriate for the thematic areas of the exhibition. In addition, participants perceived the olfactory feedback as enhancing the overall experience (M=4.55, SD=0.54).

For RQ3 (Usability and Interaction Design), the kiosk-based interaction model was evaluated positively. Participants found it easy to understand how scents could be activated (M=4.53, SD=0.73) and considered the interaction mechanism simple and intuitive (M=4.47, SD=0.70). Qualitative feedback further supported these findings, with users describing the experience as more engaging, enjoyable, and memorable due to the integration of olfactory feedback. Although reports of scent overlap and olfactory fatigue were generally limited, some participants noted residual odors between activations. These observations may be attributable to the duration of the experimental sessions, the frequency of odor activations, rapid participant turnover, and the limited ventilation time available within the *MobiCAVE* environment.

In addition to usability, participants reported highly positive perceptions regarding user experience and acceptance of the proposed system. The experience was considered enjoyable (M=4.74, SD=0.44) and memorable (M=4.64, SD=0.55), while participants expressed strong interest in using similar scent-enhanced VR applications in the future (M=4.76, SD=0.51).

The descriptive statistics for all questionnaire items are presented in Table 4, Table 5, Table 6 and Table 7. Results are grouped according to the validated questionnaire constructs described in Table 2, providing a detailed overview of participant perceptions regarding immersion, presence, realism, system validation, usability, and user acceptance.

The post-experiment questionnaire results are summarized in Table 4, Table 5, Table 6 and Table 7. Across all constructs, participants reported positive perceptions of the proposed system, particularly with respect to scent–location congruence, temporal synchronization, usability, and overall acceptance. The consistently high ratings observed across immersion, realism, and multisensory coherence suggest that participants perceived the olfactory feedback as an integrated component of the virtual environment rather than an isolated sensory effect.

The qualitative responses further supported these observations. Participants frequently described the olfactory cues as engaging, memorable, and complementary to the virtual museum experience. Several respondents highlighted the correspondence between specific scents and exhibition areas as particularly effective in reinforcing the thematic content of the environment.

#### 4.2.1. Internal Consistency of the Questionnaire Constructs

To assess the internal consistency of the composite questionnaire instrument, Cronbach’s α was computed for each of the four constructs using the post-experiment response data (N=51). For the *System Validation & Technical Efficacy* construct, the negatively worded items SYS3 (*“I noticed delays or mismatches between visual events and scents”*) and SYS5 (*“At times, the scents were too strong or too weak to be comfortable”*) were reverse-scored prior to reliability analysis. The results are summarized in Table 8.

The *User Experience & Overall Acceptance* construct demonstrated good internal consistency (α=0.776, 95% CI [0.652, 0.901]), as did *Immersion, Presence & Realism* (α=0.716, 95% CI [0.532, 0.900]), both exceeding the widely adopted threshold of α≥0.70 [32]. The *Usability & Interaction Design* construct yielded an acceptable value of α=0.513 (95% CI [0.247, 0.779]), consistent with the heterogeneous nature of usability scales that deliberately span distinct facets of interaction [28].

After reverse-scoring the negatively worded items, the *System Validation & Technical Efficacy* construct achieved a Cronbach’s alpha of α=0.590 (95% CI [0.437, 0.743]). While this value does not reach the commonly cited threshold of α=0.70, it indicates moderate internal consistency. The comparatively lower reliability may be attributable to the multidimensional nature of technical system evaluation, which incorporates diverse aspects of performance, including scent timing, comfort, perceived effectiveness, responsiveness, and overall system functionality.

Overall, the reliability analysis provides empirical support for the internal coherence of the primary experiential constructs (immersion, presence, realism, and user acceptance), while highlighting the inherent heterogeneity of composite technical evaluation instruments.

#### 4.2.2. Inter-Item Correlation Analysis

To further characterize the relationships among individual questionnaire items within each construct, Pearson product-moment correlations were computed for all item pairs. The analysis was conducted separately for each of the four constructs. Statistically significant correlations are indicated with an asterisk (* p<0.05; ** p<0.001 ) in the tables below.

Immersion, Presence, and Realism.

Within the *Immersion, Presence & Realism* construct, several item pairs showed moderate to strong positive correlations (Table 9). The strongest associations were observed between the sense of “being there” (IPR2) and scent-visual realism (IPR5; r=0.489, p<0.001), and between IPR2 and forgetting the real world (IPR4; r=0.449, p<0.001). Engagement with the virtual scenes (IPR3) correlated significantly with IPR4 (r=0.445, p=0.001) and with the conviction that scents conveyed ancient Greek settings (IPR6; r=0.468, p<0.001). Notably, the item measuring museum-like setup realism (IPR7) showed weak and non-significant associations with all other items, suggesting that perceived environmental authenticity is a partially distinct dimension from felt presence and immersion. These patterns are consistent with the established multidimensional structure of presence and immersion [24,25].

System Validation and Technical Efficacy.

Within the *System Validation & Technical Efficacy* construct, synchronization (SYS2) correlated positively and significantly with scent appearance speed (SYS6; r=0.429, p=0.002) and scent disappearance speed (SYS7; r=0.318, p=0.023), as well as with scent intensity appropriateness (SYS4; r=0.300, p=0.033) (Table 10). As expected from the reliability analysis, SYS3 (perceived delays) correlated negatively with SYS2 (r=−0.174, p=0.223) and SYS6 (r=−0.006, p=0.967), while SYS5 (intensity comfort) showed a negative association with SYS2 (r=−0.335, p=0.016). The positive correlation between SYS3 and SYS5 (r=0.375, p=0.007) indicates that participants who perceived synchronization failures were also more likely to report intensity discomfort, suggesting that these two negative indicators load on a common perceived-failure dimension. Scent appropriateness (SYS1) correlated significantly only with intensity (SYS4; r=0.290, p=0.039), reflecting the conceptual link between contextual fit and perceived intensity calibration.

Usability and Interaction Design.

Within the *Usability & Interaction Design* construct (Table 11), the strongest correlation was between ease of understanding how to trigger scents (USA1) and perceived simplicity of the kiosk interaction (USA2; r=0.518, p<0.001), confirming that these two items converge on the same learnability dimension. The remaining item pairs, including willingness to reuse the interaction scheme (USA3) and the perceived task-facilitation potential of olfactory feedback (USA4), showed weaker and non-significant associations with each other and with USA1 and USA2. This pattern suggests that usability as operationalized here spans at least two partially independent facets: learnability of the immediate interaction mechanism (USA1–USA2) and broader attitudinal acceptance of olfactory interaction (USA3–USA4).

User Experience and Overall Acceptance.

The *User Experience & Overall Acceptance* construct exhibited the most coherent inter-item correlation structure (Table 12). The strongest associations were found between overall enjoyment (UX3) and memorability (UX4; r=0.602, p<0.001), and between overall improvement of the experience (UX2) and enjoyment (UX3; r=0.600, p<0.001). Perceived memory enhancement (UX1) correlated significantly with memorability (UX4; r=0.558, p<0.001) and with overall experience improvement (UX2; r=0.515, p<0.001). Future intention to use scent-enhanced VR (UX5) showed moderate positive correlations with memorability (UX4; r=0.332, p=0.017), enjoyment (UX3; r=0.437, p=0.001), and memory enhancement (UX1; r=0.344, p=0.014). These findings support the interpretation that olfactory-enhanced VR experiences are perceived as a coherent and positive whole, in which enjoyment, memorability, and acceptance reinforce one another.

#### 4.2.3. Inferential Analysis: Effects of Prior Experience on User Outcomes

To complement the descriptive and reliability analyses, Spearman rank-order correlations were computed between individual-difference variables collected in the pre-experiment questionnaire and the two primary outcome composites: the *Immersion, Presence & Realism* (IPR) composite and the *User Experience & Acceptance* (UX) composite. Non-parametric Spearman correlations were selected because Likert-based composite scores cannot be assumed to satisfy the normality and interval-scale assumptions required by parametric alternatives. Four predictors were examined: prior CAVE experience (ordinal: Never = 0, Rarely = 1, Sometimes = 2, Often = 3), self-reported VR familiarity (1–5), self-reported scent sensitivity (1–5), and VR usage frequency (ordinal). All analyses were conducted using JASP (Version 0.19) and are summarized in Table 13.

Prior CAVE experience showed a statistically significant positive association with the UX composite (ρ=0.355, p=0.011), indicating that participants with greater prior exposure to room-scale or CAVE-type VR systems reported higher overall user experience and acceptance of the olfactory-enhanced experience. Descriptively, participants who had never used a CAVE system reported a mean UX score of M=4.47 (SD=0.43), compared with M=4.64 (SD=0.43) for those with some prior CAVE experience. Self-reported VR familiarity also correlated positively with the UX composite (ρ=0.291, p=0.038), with participants reporting higher general VR familiarity tending to express greater overall acceptance of the scent-enhanced experience.

Neither prior CAVE experience (ρ=0.178, p=0.212) nor VR familiarity (ρ=0.169, p=0.236) were significantly associated with the IPR composite, suggesting that felt immersion, presence, and realism were broadly consistent across participants regardless of their prior VR background. Similarly, self-reported scent sensitivity showed no significant association with either composite (both p>0.40), indicating that the olfactory experience was perceived positively across varying levels of individual olfactory sensitivity.

Finally, a strong positive correlation was observed between the IPR and UX composites themselves (ρ=0.556, p<0.001), indicating that participants who reported higher immersion and presence also tended to report more positive overall user experience and acceptance. This finding supports the theoretical expectation that immersive qualities and experiential satisfaction are closely coupled in multisensory VR contexts.

These results provide inferential support for the interpretation that the olfactory-enhanced experience was effective across participant backgrounds while also identifying prior immersive VR experience as a positive moderator of overall acceptance. This pattern is consistent with technology acceptance research [29], which identifies prior experience as a moderator of perceived enjoyment of novel interaction paradigms. Importantly, the absence of a significant relationship between prior experience and immersion or presence suggests that the proposed olfactory interaction design was accessible and perceptually effective even for participants encountering CAVE-type VR for the first time.

## 5. Discussion

The results demonstrate that the proposed system successfully achieves its primary objective of providing repeatable, real-time olfactory feedback suitable for CAVE-based virtual reality environments. The automated technical evaluation produced consistent results across all four odor diffusion channels, with a one-way ANOVA revealing no statistically significant differences in mean activation duration between channels (F(3,197)=0.506, p=0.678). This consistency is particularly important in multisensory VR applications, where variations in scent delivery timing could introduce unintended perceptual distortions and reduce perceived synchronization between sensory modalities. Combined with the high timing-consistency ratings reported in the post-experiment questionnaire, these findings suggest that the Arduino-Unity communication interface and relay-based activation protocol provide reliable and temporally stable scent diffusion.

The exploratory user evaluation indicates that the olfactory-enhanced experience was associated with positive participant perceptions across all evaluated dimensions, including immersion, presence, and perceived realism. It is important to note that, given the single-group post-test-only design, these results reflect participants’ subjective perceptions of the integrated multisensory experience and should not be interpreted as evidence of a causal effect of olfactory feedback in isolation. Factors such as novelty, acquiescence bias, or the inherent qualities of the virtual environment itself may have contributed to the observed ratings. Nevertheless, the consistently positive responses across all constructs, and in particular the high realism ratings, suggest that the integration of contextually aligned olfactory cues within the virtual museum environment was perceived as coherent and believable by participants.

The internal consistency analysis (Section 4.2.1) provides empirical support for the reliability of the primary experiential constructs. The *User Experience & Acceptance* and *Immersion, Presence & Realism* constructs both achieved acceptable to good Cronbach’s α values (α=0.776 and α=0.716, respectively), indicating that the adapted questionnaire items measured their intended constructs with reasonable coherence. The lower reliability observed for the *System Validation & Technical Efficacy* construct (α=0.590) may reflect the multidimensional nature of technical system evaluation, which encompasses several distinct aspects of performance, including scent synchronization, comfort, responsiveness, perceived effectiveness, and overall system functionality. While the negatively worded items (SYS3 and SYS5) were reverse-scored prior to analysis, the construct continued to exhibit only moderate internal consistency, suggesting that the scale captures multiple related but not identical dimensions of technical performance.

The inter-item correlation analysis (Section 4.2.2) further supports the internal coherence of the questionnaire constructs. Within the *User Experience & Acceptance* construct, strong positive correlations were observed among enjoyment, memorability, and overall experience improvement, suggesting that participants perceived the olfactory-enhanced experience as a coherent and positive whole. Within the *Immersion, Presence & Realism* construct, the item measuring museum-like setup authenticity (IPR7) showed weak associations with the remaining items, suggesting that perceived environmental authenticity may represent a partially distinct dimension from felt presence and immersion, a finding consistent with the established multidimensional structure of presence [24,25].

The inferential analysis (Section 4.2.3) revealed that prior CAVE experience (ρ=0.355, p=0.011) and general VR familiarity (ρ=0.291, p=0.038) were both positively associated with the UX composite, while neither predictor was significantly associated with the IPR composite. This dissociation suggests that the core perceptual qualities of the experience—immersion, presence, and realism—were broadly accessible regardless of prior VR background, whereas overall acceptance and enjoyment of the olfactory interaction paradigm were moderated by familiarity with immersive VR systems. This pattern is consistent with technology acceptance research [29], which identifies prior experience as a positive moderator of perceived enjoyment of novel interaction paradigms. Furthermore, the strong correlation between the IPR and UX composites (ρ=0.556, p<0.001) supports the theoretical expectation that immersive qualities and experiential satisfaction are closely coupled in multisensory VR contexts.

From the perspective of multisensory integration theory, the observed pattern of responses is consistent with the idea that olfactory feedback may benefit from temporal alignment and contextual congruence with visual and spatial stimuli. Such alignment is theorized to facilitate the binding of sensory signals into a unified perceptual representation [8], which may help explain the strong perceptions of environmental coherence reported by participants. In this sense, scent appeared to function not merely as an auxiliary layer but as a reinforcing modality supporting cross-modal consistency, though this interpretation remains speculative in the absence of a controlled baseline condition.

A key design characteristic of the proposed system is the interaction-driven nature of olfactory activation. The findings provide preliminary support for the proposed diegetic olfactory feedback loop as a conceptual framework for olfactory interaction design. Rather than treating scent as a continuously present atmospheric effect, the framework positions olfactory feedback as a direct consequence of user actions within the virtual environment. The consistently high ratings for scent–location congruence, synchronization, and overall realism suggest that participants perceived olfactory events as meaningful parts of the interaction process, supporting the viability of this interaction-driven approach to multisensory design.

This mechanism can be further understood through the Embodied Cognition Framework of Presence [25], which posits that presence arises from the user’s ability to construct and maintain a coherent spatial functional model of the mediated environment. In *Scentree*, action-dependent olfactory feedback may reinforce this model by validating user expectations through consistent multisensory consequences, potentially contributing to the high reported levels of presence and realism. This interpretation is further supported by enactive theories of perception [12], which emphasize that perception emerges through active exploration rather than passive reception of stimuli. From this perspective, the system enables a form of “enactive olfaction” in which scent perception is contingent on user-driven interaction.

These findings also align with embodied interaction perspectives [13], which conceptualize cognition and meaning as emerging through the coupling of action, perception, and the material environment. By integrating olfactory feedback into the diegetic feedback loop as a direct consequence of user actions, *Scentree* repositions scent from representational augmentation to situated feedback, grounding olfactory stimulation within the user’s ongoing embodied activity.

Taken together, these results indicate that interaction-driven olfactory feedback is a promising design approach for multisensory CAVE environments and that the proposed system is both technically reliable and perceptually well-received. However, all interpretations should be considered within the constraints of the present study design. Because no non-olfactory baseline condition was included, the findings constitute evidence of positive user perceptions and system feasibility rather than causal evidence of the isolated effects of olfactory feedback. Controlled comparative studies are needed to establish the independent contribution of scent to immersion, presence, and user experience in CAVE-based environments.

## 6. Limitations & Future Work

While the proposed system and its evaluation yield encouraging results, several aspects of the current work present opportunities for further development.

The present evaluation was conducted as an exploratory single-group study, in which all participants experienced the full olfactory-enhanced version of *Smelling Ancient Greece*. This design was appropriate for an initial proof-of-concept investigation, where the primary goal was to assess the feasibility of the system and gather rich descriptive data on user perceptions across multiple dimensions. However, the absence of a no-odor baseline condition means that the observed ratings reflect participants’ perceptions of the integrated multisensory experience as a whole, rather than the isolated contribution of olfactory feedback. Future studies will adopt controlled experimental designs with counterbalanced odor and no-odor conditions to enable more precise attribution of experiential effects to specific sensory modalities.

The evaluation relied on subjective self-report measures adapted from established VR and multisensory evaluation instruments, which represent the standard methodology for assessing inherently experiential constructs such as presence and immersion [24,25]. To further enrich the evaluative picture, future work will complement these measures with objective physiological indicators such as electrodermal activity, heart-rate variability, and EEG, providing a more comprehensive account of users’ perceptual and affective engagement during olfactory-enhanced VR experiences.

The participant sample, while adequate for an exploratory study of this scope, was predominantly male and drawn from a single institutional context. Expanding future evaluations to include more demographically diverse and representative samples will strengthen the generalizability of the findings across different user populations and deployment contexts.

From an engineering perspective, the current characterization of *Scentree’s* behavior within the *MobiCAVE* environment focuses on activation latency and system reliability. The spatial and temporal dynamics of scent diffusion—including concentration profiles, decay rates, and inter-zone cross-contamination—remain to be quantified. Future iterations of the system will incorporate environmental gas sensors to capture real-time concentration data, enabling the establishment of empirical diffusion curves and cross-contamination thresholds that can inform both hardware design and virtual environment layout decisions.

The current scent delivery relies on passive diffusion without active airflow management. While the spatial layout of the virtual museum was designed to mitigate cross-contamination through neutral transition zones, active airflow control would provide greater precision and reproducibility across experimental sessions. Future development will explore controllable micro-fans, directed airflow modules, and closed-loop ventilation systems driven by real-time sensor feedback, enabling finer-grained control over scent onset, intensity, and dissipation.

The diegetic olfactory feedback loop introduced in this work was formalized as a discrete-time state-transition model and supported by theoretical frameworks from enactive perception and embodied cognition. Extending this model into a computationally testable simulation capable of predicting scent propagation, user movement patterns, and multisensory timing interactions would provide a stronger formal foundation for future system design and optimization.

Finally, the present work evaluates *Scentree* as a standalone contribution without direct benchmarking against alternative olfactory VR platforms or non-olfactory VR baselines. Comparative evaluations across different delivery mechanisms, interaction paradigms, and application domains represent a natural and important direction for future research and will help situate the proposed approach within the broader landscape of multisensory VR design.

Together, these directions outline a clear and motivated trajectory for advancing olfactory interaction in CAVE-based immersive environments, building on the foundation established by the present work.

## 7. Conclusions

This paper presented *Scentree*, a custom olfactory display developed for deployment within the *MobiCAVE* environment. The system was designed to support temporally stable, repeatable, and contextually meaningful scent delivery through direct coupling between user interactions and olfactory feedback. Alongside the hardware-software implementation, the work introduced the concept of the *Diegetic Olfactory Feedback Loop*, a conceptual framework that repositions olfactory stimuli as active, interaction-driven components of the virtual environment rather than passive atmospheric enhancements.

The technical evaluation demonstrated reliable and consistent operation across all four odor diffusion channels, with no statistically significant differences in activation duration between channels (F(3,197)=0.506, p=0.678), confirming that the proposed architecture provides temporally stable scent delivery suitable for multisensory VR applications.

The exploratory user evaluation (N=51) showed that participants reported high levels of immersion, presence, realism, and overall satisfaction during the olfactory-enhanced virtual museum experience *Smelling Ancient Greece*. Internal consistency analysis confirmed acceptable to good reliability for the primary experiential constructs (Cronbach’s α=0.716 for Immersion, Presence & Realism; α=0.776 for User Experience & Acceptance). Inter-item correlation analysis further supported the coherence of the questionnaire instrument, with particularly strong associations observed among enjoyment, memorability, and overall experience improvement. Inferential analysis revealed that prior CAVE experience (ρ=0.355, p=0.011) and VR familiarity (ρ=0.291, p=0.038) were positively associated with overall user acceptance, while perceived immersion and presence were consistently high across all participant backgrounds, suggesting that the olfactory interaction design was perceptually accessible regardless of prior VR experience. It should be noted that the single-group, post-test-only design does not permit causal conclusions regarding the isolated contribution of olfactory feedback; the findings are therefore interpreted as evidence of positive user perceptions and system feasibility rather than causal evidence of olfactory effects on immersion or presence.

From a theoretical perspective, the findings are consistent with established frameworks of multisensory integration, embodied cognition, enactive perception, and embodied interaction. By linking olfactory stimuli directly to user actions and environmental context, *Scentree* extends olfactory feedback beyond ambient scent delivery and supports a more active and meaningful form of multisensory engagement.

Overall, this work contributes three primary outcomes: a reproducible, low-cost olfactory feedback architecture suitable for CAVE environments; a conceptual framework for interaction-driven scent integration; and an empirical, psychometrically grounded evaluation of olfactory-enhanced VR within a shared immersive environment. Together, these contributions provide a foundation for future research on multisensory interaction design in CAVE-based systems. Future work should prioritize controlled comparative studies with odor and no-odor conditions, physiological and behavioral outcome measures, and investigations of collaborative and task-oriented applications of interaction-driven olfactory feedback.

## Figures and Tables

**Table 1:** Hardware Components.

Component	Quantity	Specifications	Purpose
Arduino Uno R3	1	ATmega328P, 5 V, USB Serial	Receives signals from Unity (version 2022.3.60f1 LTS) and controls the relay module
Four Channel Relay Module	1	COM/NO Contacts, 5 V	Emulates button presses
Ultrasonic piezoelectric transducer Module	4	5 V, Ceramic Atomizer, Onboard control PCB	Generates scented mist
P iezoelectric transducer Driver Board	4	Integrated Tactile Button Pads	For modifying their button pads
Plastic container	4	Plastic, 50–75 mL	Stores scented liquid
Multi-port USB HUB	1	5 V Output	Powers the piezoelectric transducer Driver Boards
External Power Supply	1	5 V DC output, USB-A Ports	Provides portability and powers the USB Hub
USB A to MicroUSB Cable	4	5 V Power Delivery	Used to connect with the piezoelectric transducer PCB
USB A to USB B Cable	1	Default Arduino Cable	Connects Arduino to PC
Dupont Jumper Wire Kit	1	Flexible	Wires the Arduino with the relay module
Jumper Wires for Soldering	8	Insulated Copper	Solder to button pads for modification

**Table 2:** Survey Items.

Construct	Survey Item	Code	Source
Immersion, Presence & Realism	I felt immersed in the virtual museum environment.	IPR1	[24]
	I had the feeling of “being there” in the virtual exhibition.	IPR2	[25]
	I felt engaged with what was happening in the virtual scenes.	IPR3	[24]
	I often forgot about the real world around me while I was in the virtual museum.	IPR4	[25]
	The combination of visuals and scents made the experience feel realistic.	IPR5	[27]
	The scenes and scents together gave me a convincing impression of ancient Greek settings.	IPR6	[27]
	The setup of the virtual exhibition matched what I would expect in a real museum.	IPR7	[26,27]
System Validation & Technical Efficacy	The experience’s scents seemed appropriate for the scenes that were shown.	SYS1	[31]
	The release of scents was well synchronised with what was happening in the virtual scene.	SYS2	[30,31]
	I noticed delays or mismatches between visual events and scents.	SYS3	[31]
	The intensity of the scents was appropriate for the experience.	SYS4	[31]
	At times, the scents were too strong or too weak to be comfortable.	SYS5	[31]
	The scent appeared quickly when the event started.	SYS6	[31]
	The scent disappeared quickly enough before the next one.	SYS7	[31]
Usability & Interaction Design	It was easy to understand how to trigger the scents during the experience.	USA1	[28]
	Interacting with the kiosks and pressing the key to release scents felt simple and intuitive.	USA2	[28]
	I would feel comfortable using this interaction scheme in a similar experience again.	USA3	[29]
	The addition of smells can make tasks easier (e.g., within a puzzle game setup).	USA4	[30]
User Experience & Acceptance	The addition of scents helped me memorize information better.	UX1	[8,30]
	Overall, the addition of scents improved my virtual museum exhibition experience.	UX2	[31]
	The experience of exploring the virtual museum with scents was enjoyable.	UX3	[31]
	The scents contributed to making the experience memorable for me.	UX4	[8]
	I would like to use similar scent-enhanced VR experiences in the future.	UX5	[29]

**Table 3:** Performance Results.

Channel	Trials	Mean (ms)	SD (ms)
1	50	6800.6	0.72843136
2	50	6800.74	0.75078191
3	51	6800.66667	0.68313005
4	50	6800.76	0.74395523
**Total**	201	6800.692	0.72414

**Table 4:** Immersion, Presence and Realism Questionnaire Results.

Code	Survey Item	Mean	SD
IPR1	I felt immersed in the virtual museum environment.	4.14	0.77
IPR2	I had the feeling of “being there” in the virtual exhibition.	4.02	0.83
IPR3	I felt engaged with what was happening in the virtual scenes.	4.27	0.63
IPR4	I often forgot about the real world around me while I was in the virtual museum.	3.39	1.09
IPR5	The combination of visuals and scents made the experience feel realistic.	4.49	0.61
IPR6	The scenes and scents together gave me a convincing impression of ancient Greek settings.	4.27	0.75
IPR7	The setup of the virtual exhibition matched what I would expect in a real museum.	4.43	0.60

**Table 5:** System Validation and Technical Efficacy Results.

Code	Survey Item	Mean	SD
SYS1	The experience’s scents seemed appropriate for the scenes that were shown.	4.25	0.84
SYS2	The release of scents was well synchronised with what was happening in the virtual scene.	4.47	0.73
SYS3	I noticed delays or mismatches between visual events and scents.	1.90	1.20
SYS4	The intensity of the scents was appropriate for the experience.	4.35	0.68
SYS5	At times, the scents were too strong or too weak to be comfortable.	1.98	1.10
SYS6	The scent appeared quickly when the event started.	4.11	1.16
SYS7	The scent disappeared quickly enough before the next one.	3.66	1.29

**Table 6:** Usability and Interaction Design Results.

Code	Survey Item	Mean	SD
USA1	It was easy to understand how to trigger the scents during the experience.	4.52	0.73
USA2	Interacting with the kiosks and pressing the key to release scents felt simple and intuitive.	4.47	0.70
USA3	I would feel comfortable using this interaction scheme in a similar experience again.	4.72	0.66
USA4	The addition of smells can make tasks easier (e.g., within a puzzle game setup).	4.21	0.92

**Table 7:** User Experience and Acceptance Results.

Code	Survey Item	Mean	SD
UX1	The addition of scents helped me memorise information better.	4.11	0.84
UX2	Overall, the addition of scents improved my virtual museum exhibition experience.	4.54	0.54
UX3	The experience of exploring the virtual museum with scents was enjoyable.	4.74	0.44
UX4	The scents contributed to making the experience memorable for me.	4.64	0.55
UX5	I would like to use similar scent-enhanced VR experiences in the future.	4.76	0.51

**Table 8:** Cronbach’s α per questionnaire construct (95% CI).

Construct	α	95% CI Lower	95% CI Upper
Immersion, Presence & Realism	0.716	0.532	0.900
System Validation & Technical Efficacy	0.590	0.437	0.743
Usability & Interaction Design	0.513	0.247	0.779
User Experience & Overall Acceptance	0.776	0.652	0.901

**Table 9:** Pearson correlations among Immersion, Presence & Realism items. * p<0.05; ** p<0.001.

	IPR2	IPR3	IPR4	IPR5	IPR6	IPR7
**IPR1**	0.243	0.247	0.312 *	0.235	0.106	0.042
**IPR2**		0.366 *	0.449 **	0.489 **	0.406 *	0.180
**IPR3**			0.445 *	0.367 *	0.468 **	0.153
**IPR4**				0.185	0.353 *	−0.019
**IPR5**					0.485 **	0.012
**IPR6**						0.086

**Table 10:** Pearson correlations among System Validation & Technical Efficacy items. * p<0.05; ** p<0.001. Dagger (†) denotes items that correlated negatively with the scale.

	SYS2	SYS3 †	SYS4	SYS5 †	SYS6	SYS7
**SYS1**	0.223	−0.034	0.290 *	−0.230	0.132	0.190
**SYS2**		−0.174	0.300 *	−0.335 *	0.429 *	0.318 *
**SYS3**			0.043	0.375 *	−0.006	0.069
**SYS4**				−0.201	0.273	0.315 *
**SYS5**					−0.076	0.094
**SYS6**						0.454 **

**Table 11:** Pearson correlations among Usability & Interaction Design items. ** p<0.001.

	USA2	USA3	USA4
**USA1**	0.518 **	−0.065	0.272
**USA2**		0.111	0.241
**USA3**			0.163

**Table 12:** Pearson correlations among User Experience & Overall Acceptance items. * p<0.05; ** p<0.001.

	UX2	UX3	UX4	UX5
**UX1**	0.515 **	0.407 *	0.558 **	0.344 *
**UX2**		0.600 **	0.455 **	0.114
**UX3**			0.602 **	0.437 *
**UX4**				0.332 *

**Table 13:** Spearman rank correlations between pre-experiment individual-difference variables and post-experiment outcome composites (N=51). * p<0.05.

Predictor	IPR Composite		UX Composite	
	ρ	p	ρ	p
Prior CAVE experience	0.178	0.212	0.355	0.011 *
VR familiarity	0.169	0.236	0.291	0.038 *
Scent sensitivity	0.094	0.510	0.120	0.403
VR usage frequency	0.008	0.954	0.193	0.174

## Data Availability

The original data presented in the study are openly available in Github at: https://github.com/VasilisVasOg/Scentree_Project, accessed on 14 June 2026.

## Abbreviations

The following abbreviations are used in this manuscript:

ANOVA	Analysis of Variance
ASCII	American Standard Code for Information Interchange
BLTE	Bluetooth Low Energy
CAVE(s)	Cave Automatic Virtual Environment(s)
CI	Confidence Interval
CSV	Comma-Separated Values
DC	Direct Current
DST(s)	Digital Scent Technology/Technologies
EEG	Electroencephalogram
GPS	Global Positioning System
HMD(s)	Head-mounted Display(s)
IPQ	Igroup Presence Questionnaire
M	Mean
N	Sample size
OVR	Olfactory Virtual Reality
PCB	Printed Circuit Board
PQ	Presence Questionnaire
PRS	Perceived Recovery Scale
ROS	Rehabilitation Outcome Scale
SD	Standard deviation
SUS	System Usability Scale
TAM	Technology Acceptance Model
USB	Universal Serial Bus
VR	Virtual Reality
